# The Permanent Cure of Hernia

**Published:** 1882-01

**Authors:** W. H. Heath

**Affiliations:** Assistant Surgeon U. S. Marine Hospital Service


### BUFFALO

### Medical and Surgical Journal.

Vol. XXI. JANUARY, 1882. No. 6.

### Original Communications.

### The Permanent Cure of Hernia.

By W. H. Heath, M. D., Assistant Surgeon U. S. Marine Hospital Service.

The interest manifested by those of the profession I have been brought in contact with here, the several extracts from, and reference to the article which appeared in the May number of this journal, suffices to show that even a meagre contribution to the subject is considered worthy of attention.

To those who may avail themselves of the method of treatment known as the Heatonian, there are some points of practical importance influencing the results, which I consider of sufficient moment to speak of again, even at the expense of repetition. The selection of fit cases to operate upon is most essential. I know of no procedure in surgery where this is of any greater necessity, yet so little said about, and I am confident many failures can be traced to this neglect, where cause is attributed to the method itself. A first or second failure to a beginner would be extremely unfortunate and disheartening, and probably cause a hasty and erroneous opinion of the value of the procedure. It is not an operation of emergency, or at any time of necessity to life; therefore the selection of proper cases is perfectly feasible. Some cases with contra-indications present can be suitably treated with a view of removing them, to be fit for operating at a later period; others should never be subjected to it at all.

No case should be attempted while suffering from any condition likely to interfere with perfect rest in the recumbent position. A cough due to either chronic or acute bronchial difficulty, would, by disturbing the abdominal contents, force the protrusion against and into the inguinal canal; any trouble of the digestive tract causing vomiting, accumulation of flatus in larger quantity, or looseness of the bowels, would be equally detrimental. It is necessary to keep the bowels quiet for four or five days at least; administration of opium for this purpose should be done with care, not to provoke nausea.

Chronic cystitis, or irritability of the bladder from any cause, stands in the same relation. Epilepsy, where the attacks are of frequent occurrence, is absolutely against success, one seizure would undo all that had been gained. Albuminuria is a contra-indication, as in all surgical operations. In the one case where I resorted to this method upon a man with albumen in his urine, pus promptly formed; the abscess, however, healed after many months.

With children of a restless or very nervous disposition, action had better be postponed for some years until they are more amenable. Very young children are not fit candidates, nor the aged or feeble, for reasons too obvious to mention. The best period of life I should say would be from ten to thirty years of age, and the most promising class of hernia, the oblique reducible, and particularly the congenital variety; femoral hernia are the least promising and accompanied by more danger.

When the parts are inflamed from wearing a truss, or from any cause, it should first be allayed; in brief, the patient should be in general good health and condition.

These considerations are of practical importance, as likely to affect the result, and I trust will be borne in mind by all who intend operating by this method.

The manner in which a cure is brought about is apparently not clear to some. In the first place *the sac is not injected*, nor does it take part at all in the procedure; when possible it should be reduced along with the hernia; its presence in the canal, however, does not interfere with a successful result; it is the fibrous structures, and they alone, that are concerned in the matter. Nor is the cure effected by a glueing together of the edges of the ring with lymph, as has been supposed; on the contrary the hernial passage is *obliterated* throughout its extent by thickening and contraction of the fibrous structures which surround it, the thickening depending upon the production or effusion of plastic lymph, largely interstitial, rendering the parts more rigid, less yielding, while the contraction which occurs and is peculiar, and has much to do in bringing about the result, depends on the anatomical arrangement of the fibres.

The canal is not actually glued together at its edges, nor in fact anywhere; it is an obliteration of it by a shrinking and thickening of the structures which enter into its formation, and which are fibrous. It is in this way that the parts are restored to exercise their functions, and in principle and pathology is distinct from other methods.

This thickening from effusion of lymph, contraction, and obliteration is a reality, and can be detected and its gradual formation watched and demonstrated by carefully examining the parts at varying intervals after they have been irritated.

Some hernia require a second or third injection to bring about this condition, especially in hernia of some time standing and with large patulous rings. After the lapse of two or three days, if there is but a slight amount of tenderness or firm pressure, and examination reveals the fact that but a slight impression has been made upon the structures, they should be thoroughly wetted again as in the first instance. This very generally has the desired effect, but should it not, it may be again repeated. The danger of abscess is not great, the tissues involved are not vascular, being maintained more particularly by nutritive juices, and therefore less liable to inflame; besides the mild character of the irritant has to do with lessening the danger of this sort.

The permanency of the condition brought about at once comes up as a question of practical import. If these results are but transient, the lymph becoming absorbed after a period, allowing the hernia to descend again, the method at its best is but palliative, a temporary one, the transient benefit scarcely warranting the patient subjecting himself to the inconvenience of having the operation performed. Disappointment from other operations in this respect have been so constant that foregone conclusions are generally formed, and especially to this method which claims so much and is apparently so simple. In evidence of the duration of these results, we can refer to the numerous examinations of patients at periods varying from months to years after being operated on. The class of patients my operations have mostly been upon pass from my observation after a few months. I have, however, had the opportunity of examining several after six months; the last one after a period of nine months, a man who had been actively engaged during that time, in his case there was no evidence of any retrograde change going on. Heaton and Warren report cases examined after many years and found to be permanently cured. I may say that in all cases examined by me, that had stood the test of six months, I could discover no evidence of absorption of lymph or returning hernia.

Besides this, which may be considered the "proof of the pudding," we have the well-established pathological fact that fibrous structures, generally, when subjected to inflammatory action are apt to be permanently altered, that the changes which occur under the condition of inflammation are likely to remain; thickening of periarticular structures and of the heart valves after endocarditis are examples of this principle. Post-mortem examination of this region, at any time after operating, would be highly interesting and valuable in this connection, but as death is not a part of the history of this method, and as patients after a time, generally, pass from view of the operator, it rarely falls to the lot of any one to make such observations. Heaton mentions one case where he had the opportunity of examining the parts many years after making a permanent cure, and according to him they were so restored, that it was difficult to conceive a hernia had ever existed. Without, therefore, discussing further the possibility or impossibility of absorption taking place, I think we are justified in considering that even if it does go on at all, it does so, in so slow a manner as practically not to interfere with the desired result.

Another point, and upon which I have been questioned, is where the instrument is introduced and what instrument is the best.

The object is to insert the needle of the instrument into the inguinal canal with as little injury to the tissues as possible and without any to the contents of the canal or abdomen. The most direct way is therefore the best, and the first point is to locate the entrance to the canal,—the external abdominal ring, upon the surface directly over it. This is accomplished by first defining it with a finger of the right hand, invaginating the scrotum, in a manner similiar to that followed in examining for the existence of hernia, and then transferring the location of it to a finger of the left hand from the surface, thus leaving the right hand free to manipulate the instrument. After the right-hand finger has clearly made out the ring, the finger of the left should rapidly be pushed into it, carrying with it the intervening tissues, and as this is being done the right hand is withdrawn, so that the exit abdominal ring is localized from the surface and by the left hand. The contents of the canal—the cord—is protected from injury by being pushed towards the median line out of the way, and retained in that position by the left hand finger when it descends into the ring from the surface.

The abdominal contents can only be injured by penetrating the transversalis fascia, which, while it would not be a fatal accident would be very unfortunate. Such a circumstance could only happen by carelessness and is best averted by great gentleness in handling the needle after it is in the canal, and remembering there is an alteration in the relation of the parts, in all hernia of any duration, viz., that the internal ring is dragged down towards the external, and the canal is consequently shortened and its direction less oblique. The needle, when it is passed in, is not pushed through either pillar of the external ring, on the contrary it should enter the aperture clear and free, though close to the external pillar, it should be introduced directly from the surface alongside of the finger which is pressed into the ring and which serves as a guide for it, this is the most direct and the easiest way; it is never passed in from below through the invaginated scrotum.

After penetrating the tissues and entering the aperture its point will be found free to move in all directions, at this time the finger should again be invaginated through the scrotum and the exact position of the needle verified before its direction is changed and it is carried on into the canal.

The fluid is deposited from the instrument as it is gradually being withdrawn, care being taken to wet thoroughly all the structures from the one end of the canal to the other, and particularly all around the external opening.

For a long time I was in the habit of using Dr. DeGarmo's (of New York) needle, which is a sharp-pointed instrument, arranged with an attachment to sheath the point after it has penetrated the tissues so that it is carried forward into the canal, blunt, to prevent injury. The syringe is a small one, holding about 20 m, the piston working on a screw to gradually deposit its contents.

This very convenient and ingenious little instrument is easily managed, but is not altogether a safe one, the sharp point being liable at any time, even with the greatest care, to wound a vessel in its passage, or puncture the cord, which is frequently flattened out from pressure and difficult to keep entirely out of the way of danger.

Recently I have been using a syringe and needle devised by Dr. Warren, of Boston, which I consider the safest and best. The needle, instead of being round, is flattened and somewhat oval, has a spiral twist to it, and also many more openings for the fluid to escape; the point is also blunted. The barrel and piston are so arranged with a spring on the latter, as to retain the fluid in the chamber under considerable pressure, which at the proper time is allowed to escape by opening a valve, which causes the fluid to be projected with some force through the apertures on each side of the needle. The advantages of the instrument are less liability of injury to vessels or cord, and the structures are more thoroughly wetted by the fluid which is sprayed out, rather than dropped as in the DeGarmo needle.

